# Diet with a combination of high protein and high total antioxidant capacity is strongly associated with low prevalence of frailty among old Japanese women: a multicenter cross-sectional study

**DOI:** 10.1186/s12937-017-0250-9

**Published:** 2017-05-12

**Authors:** Satomi Kobayashi, Hitomi Suga, Satoshi Sasaki, the Three-generation Study of Women on Diets and Health Study Group

**Affiliations:** 1 Department of Social and Preventive Epidemiology, School of Public Health, the University of Tokyo, 7-3-1 Hongo, Bunkyo-ku, Tokyo, 113-0033 Japan; 2 Department of Social and Preventive Epidemiology, Graduate School of Medicine, the University of Tokyo, Tokyo, Japan

**Keywords:** Protein, Total antioxidant capacity, Frailty, Brief-type self-administered diet history questionnaire, Old Japanese women

## Abstract

**Background:**

The intake of protein and antioxidants has been inversely associated with frailty, individually. However, to our knowledge, no study has evaluated these associations in considering antioxidants or protein intakes as respective confounders. Further, the cooperative effect of dietary protein and antioxidants on frailty has not been investigated. Therefore, we examined the association of high protein and high dietary total antioxidant capacity (TAC) with frailty under the adjustment for dietary TAC or protein intake, respectively. The association between the combination of high dietary protein and high dietary TAC and frailty was also investigated.

**Methods:**

A total of 2108 grandmothers or acquaintances of dietetic students aged 65 years and older participated in this cross-sectional multicenter study conducted in 85 dietetic schools in Japan. Dietary variables, including protein intake, and dietary TAC were estimated from a validated brief-type self-administered diet history questionnaire. Frailty was defined as a score of three or more points obtained from the following four components: slowness and weakness (two points), exhaustion, low physical activity, and unintentional weight loss.

**Results:**

Median (interquartile range) age of the present subjects was 74 (71–78) years. Multivariate adjusted ORs (95% CIs) for frailty in the highest compared to the lowest tertile were 0.66 (0.49, 0.87) for total protein intake (*P* for trend = 0.003) and 0.51 (0.37, 0.69) for dietary TAC (*P* for trend <0.0001) after adjustment for dietary TAC or total protein intake, respectively. The OR of frailty for the group with both the highest tertiles of total protein intake and dietary TAC was markedly lower (multivariate adjusted OR [95% CIs]: 0.27 [0.16, 0.44]; *P* <0.0001) compared to the group with the lowest tertile of protein intake and the lowest tertile of dietary TAC.

**Conclusions:**

Both protein intake and dietary TAC were independently inversely associated with frailty among old Japanese women. Further, a diet with the combination of high dietary protein and high dietary TAC was strongly inversely associated with the prevalence of frailty in this population. To select food combinations that allow for an increase of both protein and antioxidants in diet according to the local food culture and dietary habits may be an effective strategy for frailty prevention.

## Background

Population aging is continuing worldwide [1]. People with frailty, a health status among older populations characterized by low physiological reserves and vulnerability to several stressors [2], are assumed to be increasing in the world, because the prevalence of frailty increases with age [3, 4]. Frail individuals have higher risks of subsequent disability, falls, hospitalization, and death than those who are not frail [2, 5–7]. Therefore, the prevention of frailty is important for minimizing these adverse health outcomes and for meeting the challenge of successful aging in rapidly aging countries, including Japan [8].

Poor nutritional status is assumed to be one of the important modifiable risk factors for frailty [9–12]. Previous observational studies have suggested that adequate intakes of macronutrients and micronutrients may reduce the risk of frailty [9–12]. For example, some cross-sectional [13–15] and prospective [16, 17] studies have shown that higher protein intake was associated with lower risk of frailty. The intake of antioxidant nutrients, such as vitamin E and vitamin C [14] or resveratrol [18], and dietary total antioxidant capacity (TAC) [19] was also inversely associated with frailty. However, none of these studies considered other targeted dietary variables assumed to be associated with frailty as confounders. The independent effects of a high-protein or high-antioxidant diet on frailty with simultaneous consideration of each other in a statistical model have not been examined yet.

Since people do not consume single nutrients but instead meals containing a combination of foods with a wide range of nutrients, investigating the influences of nutrient combinations on frailty may be more useful than analyzing the influences of single nutrients when developing a strategy of frailty prevention. Unfortunately, previous studies have not identified effective nutrient combinations that prevent frailty [9, 11]. The cooperative effect of dietary protein and antioxidants on frailty has also not been examined yet.

Further studies that identify the effect of single and combined dietary intake on frailty are needed in order to develop effective and general strategies for frailty prevention. Here, we investigated the independent association between protein intake or dietary TAC and frailty among old Japanese women under the adjustment for dietary TAC or protein intakes, respectively. Further, the effect of a diet combining high protein and high dietary TAC on frailty was also evaluated to investigate the cooperative association of protein and antioxidants to frailty.

## Methods

### Procedure

This cross-sectional study was based on data from the Three-generation Study of Women on Diets and Health. A detailed description of the study design and survey procedure has been published elsewhere [13, 19]. Briefly, two questionnaires for diet and lifestyle were distributed to a total of 7016 dietetic freshmen students in April 2011 or 2012. The students were also requested to directly distribute the questionnaires to their mothers and grandmothers or 65–to 89-year-old female acquaintances and invite them to join the study. The protocol of the study was approved by the Ethics Committee of the University of Tokyo Faculty of Medicine (approval number: 3249; approved on November 29, 2010). Written informed consent was obtained from all participants. The subjects for the present study were the participants of the old women for the grandmothers’ generation.

### Dietary assessment

Dietary habits during the preceding month were assessed using a previously validated, brief-type self-administered diet history questionnaire (BDHQ) designed to assess an individual’s habitual dietary intake [20, 21]. Details of the BDHQ’s structure, method of calculating dietary intake, and validity for commonly studied food and nutrient intakes have been published elsewhere [20, 21]. Estimates of the daily intake for 58 foods, energy, and selected nutrients, including protein, were calculated based on the Standard Tables of Food Composition in Japan [22]. Protein from fish and shellfish, meat, eggs, and dairy products was included in animal protein. Protein from cereals, pulses, potatoes, confectionaries, fruits, vegetables, alcoholic beverages, and non-alcoholic beverages was included in plant protein. Pearson’s correlation coefficients of protein intake between that from the 16-d dietary record and that from the BDHQ in 92 women aged 31–69 was 0.35 [21]. Dietary TAC was calculated using the answers of the BDHQ and the TAC value we assigned to each food item in the BDHQ [19]. To develop the TAC database for the BDHQ, we searched for analytical values by PubMed database. When the values could not be obtained, substituted values (analytical values of similar foods), or calculated values (calculated analytical values of ingredient food) was used. Dietary TAC was estimated based on intake and the TAC value of each food [19]. Although the validity of dietary TAC estimated from the BDHQ has not been evaluated, the previous validation study of the BDHQ among 92 adult women for foods and nutrients reported that Spearman’s correlation coefficients for some food groups, which are the main contributors of dietary TAC [19], were 0.64 for green tea, 0.77 for coffee, 0.55 for total vegetables, and 0.41 for fruits [20] and Pearson’s correlation coefficients for some antioxidant nutrients were 0.57 for β-carotene, 0.66 for vitamin C, and 0.48 for α-tocopherol [21]. In the present study, we used the value assayed by oxygen radical absorbance capacity (ORAC) as the TAC value since ORAC value was the most strongly associated with frailty in our previous study [19]. Meanwhile, we confirmed the association between dietary TAC and frailty by using other dietary TAC value we previously evaluated, namely ferric reducing ability of plasma (FRAP), Trolox equivalent antioxidant capacity (TEAC), and total radical-trapping antioxidant parameter (TRAP) [19]. Although dietary supplement use was queried in the lifestyle questionnaire, intake from supplements was not included in the calculation for nutrient intakes and dietary TAC due to the lack of a reliable composition table of dietary supplements in Japan. The supplement use was treated as confounding factors.

### Frailty

Although frailty was operationally defined by Fried et al. [2] to include the measures of walking speed for slowness and grip strength for weakness, we did not obtain these measures in our study, but rather used the modified definition developed by Woods et al. [5]. Frailty was assessed using the following four components: 1) slowness and weakness (physical functioning scale of the Japanese version short-form 36-item health survey [SF-36] < 75) [23–25]; 2) exhaustion (vitality scale of SF-36 < 55); 3) low physical activity (those in the lowest quartile); and 4) unintentional weight loss (self-reported unintentional weight loss in the previous one year > 5%). Physical activity was calculated as the average metabolic equivalent-hours, on the basis of the self-reported duration of five activities (walking, bicycling, standing, running, and high-intensity activities) and sleeping and sitting hours over the preceding month, and the metabolic equivalent (MET) value assigned to each activity [26].

Slowness and weakness was scored as two points, and the other components as one point each. Total frailty score was the sum of all available scores (0–5), with those subjects with a total score ≥ 3 defined as frail [5].

### Other variables

Body mass index (BMI) was calculated as current body weight (kg) divided by the square of body height (m). Residential area was grouped into six regions (Hokkaido and Tohoku, Kanto, Hokuriku and Tokai, Kinki, Chugoku and Shikoku, and Kyushu) and also into three categories according to population size (city with a population ≥ 1 million, city with a population < 1 million, and town and village). The subject also reported whether she was living alone, as well as her marital status (single, married, widowed, or separated), education (≤ junior high school and others, high school, and ≥ college), current smoking status, alcohol drinking, dietary supplement use, and history of chronic diseases. A history of chronic disease, including stroke, myocardial infarction, hypertension, diabetes, and chronic rheumatism, with which the proportions of the subject were different between frail group and non-frail group, was considered as an indicator of past health status. Since the proportions of the subject with other diseases, i.e. any cancers and hepatic disease, were not different between these groups, we did not include these diseases as chronic disease. Depression symptoms were assessed using the Center for Epidemiologic Studies Depression (CES-D) scale [27, 28] incorporated in the lifestyle questionnaire, with subjects with a CES-D score ≥ 16 considered to have depression symptoms.

### Statistical analysis

All dietary variables were adjusted for energy by the residual method using a linear regression model [29] and the density method as a percentage of daily energy intake for energy-containing nutrients or per 1000 kcal of daily energy intake for non-energy-containing nutrients, foods, and dietary TAC. The characteristics of the subjects with and without frailty were compared using Mann–Whitney signed-rank test for continuous variables or the chi-square test for categorical variables. We used the non-parametric test because the result of Kolmogorov-Smirnov test for normality showed that all continuous variables had non-normal distribution.

The subjects were divided into tertiles according to protein intake and dietary TAC adjusted by the residual method. Odds ratios (ORs) and 95% confidence intervals (CIs) of each protein and dietary TAC for frailty were calculated by different logistic regression model after adjusting for potential confounding factors. The initial model was a crude model into which covariates were added using forward selection method. Final multivariate models used age (y, continuous), BMI (kg/m^2^, continuous), residential block (six blocks), size of residential area (three areas), living alone (yes or no), current smoking (yes or no), alcohol drinking (yes or no), dietary supplement use (yes or no), history of chronic disease (yes or no), depression symptoms (yes or no), and energy intake (kcal/d, tertiles) as potential confounding factors. Other variables, namely survey year (2011 or 2012), marital status (four categories), and education (three categories), were not included in the models, because these variables had no influence on the relationship between dietary variables and frailty (*P >*0.10). We further adjusted for other dietary variables (i.e., dietary TAC for total protein; plant protein and dietary TAC for animal protein; animal protein and dietary TAC for plant protein; and total, animal, and plant protein for dietary TAC [tertile]). In this examination, each dietary variable was input to the one model, e.g., to examine the association between total protein and frailty with the adjustment for dietary TAC, we input total protein intake and dietary TAC into the model, simultaneously. The subjects were further divided into nine (3 × 3) groups defined by the combination of total protein intake (tertile) and dietary TAC (tertile). Adjusted odds ratios of frailty for these nine groups were also calculated using the same model. The dietary intakes in three groups of the lowest tertile for both protein intake and dietary TAC (P1A1), intermediate tertile for them (P2A2) and highest tertile for them (P3A3) were compared by Kruskal-Wallis test. These dietary variables were selected to describe comprehensive dietary intake among the present subjects. An analysis using dietary variables adjusted by the density method was also conducted.

All statistical analyses were performed with SAS statistical software, version 9.4 (SAS Institute Inc., Cary, NC, USA). All reported *P* values were two-tailed, with a *P* value of < 0.05 considered statistically significant.

## Results

A total of 2332 women in the grandmothers’ generation (33.2%) answered both questionnaires. We excluded those subjects who lived in eastern Japan and answered questionnaires in 2011 (*n =* 47), because of the Great East Japan Earthquake in March 2011. We also excluded a subject (*n =*1) in an institution because of standardization, where the response rate for participating home was extremely low (4%) than all the other institutions (35-100%). Further, we excluded subjects whose age, height, weight, or residential area was missing (*n =* 20); those aged < 65 years (*n =* 65); and those with a reported energy intake less than half of the energy requirement for the lowest physical activity category according to the Dietary Reference Intakes for Japanese, 2010 (<725 kcal/d; *n =* 14) [30] or those with an intake more than 1.5 times of the energy requirement for the highest physical activity category (> 3300 kcal/d; *n =* 32). We further excluded those with Parkinson’s disease (*n =* 8), chronic kidney disease (*n =* 13), those who were unable to walk (*n =* 20; to avoid misclassification of frailty), and those with missing information on the variables used for the purpose of multivariate analysis (*n =* 4). The final sample thus comprised 2108 women aged 65–94 years. The subject excluded from the present study was significantly younger, and had lower proportions of living alone and a history of chronic disease than the study population (data not shown).

Median age (interquartile range [IQR]) of the study population was 74 (71–78) years and median BMI was 22.5 (20.6–24.7) (Table 1). A total of 481 women (22.8%) were classified as frail. Compared with the non-frail group, the frail group was significantly older, had a higher BMI and more current smokers, higher proportions of a history of chronic disease and depression symptoms, and fewer alcohol drinkers and supplement users. Median (IQR) intake (and contribution to energy values) of protein were 73.1(65.0–81.4) g/d (16.7 [14.7–18.8] %) for total protein, 42.0 (33.7–51.8) g/d (9.5 [7.4–11.9] %) for animal protein, and 30.3 (27.7–33.2) g/d (7.0 [6.4–7.6] %) for plant protein (Table 2). Protein intake per body weight (BW) was 1.43 (1.22–1.67) g/kg BW/d. Median (IQR) (energy-adjusted value) dietary TAC was 20.2 (15.7–25.0) mmol TE/d (11.7 [9.0–15.1] mmol TE/1000 kcal). The Spearman’s correlation coefficients between proteins and dietary TAC were 0.07 for total protein, −0.03 for animal protein, and 0.24 for plant protein, and that between animal protein and plant protein was −0.34 (data not shown). Energy intake among the frail subjects was significantly lower than among the non-frail ones. Protein intake and dietary TAC in the frail group were significantly lower than those in the non-frail group. The median values of frail group to those of non-frail group were 96, 94, and 97% for total protein, 93 and 92% for animal protein, 99 and 99% for plant protein, and 87 and 89% for dietary TAC.

**Table 1:** Basic characteristics of 2108 old Japanese women categorized with and without frailty^a^

	Total (*n =* 2108)				No frailty^b^ (*n =* 1627)				Frailty^b^ (*n =* 481)				*P* ^c^
	Median, IQR or n (%)				Median, IQR or n (%)				Median, IQR or n (%)				
Age, years	74	71	-	78	74	70	-	77	77	74	-	81	<0.0001
Body height, cm	150.0	147.0	-	154.0	150.2	147.7	-	154.0	150.0	145.0	-	153.0	<0.0001
Body weight, kg	51.0	46.0	-	56.0	51.0	46.1	-	56.0	51.0	45.0	-	57.0	0.71
Body mass index, kg/m^2^	22.5	20.6	-	24.7	22.4	20.5	-	24.4	22.8	20.8	-	25.4	0.02
Residential block, n (%)													0.38
Hokkaido and Tohoku	193 (9.2)				142 (8.7)				51 (10.6)				
Kanto	526 (25.0)				404 (24.8)				122 (25.4)				
Hokuriku and Tokai	510 (24.2)				410 (25.2)				100 (20.8)				
Kinki	262 (12.4)				199 (12.2)				63 (13.1)				
Chugoku and Shikoku	342 (16.2)				265 (16.3)				77 (16.0)				
Kyushu	275 (13.1)				207 (12.7)				68 (14.1)				
Size of residential area, n (%)													0.26
City with a population ≥1 million	274 (13.0)				204 (12.5)				70 (14.6)				
City with a population <1 million	1598 (75.8)				1247 (76.6)				351 (73.0)				
Town and village	236 (11.2)				176 (10.8)				60 (12.5)				
Living alone, n (%)													0.30
No	1759 (83.4)				1365 (83.9)				394 (81.9)				
Yes	349 (16.6)				262 (16.1)				87 (18.1)				
Marital status, n (%)													<0.0001
Single	4 (0.2)				3 (0.2)				1 (0.2)				
Married	1275 (60.5)				1035 (63.6)				240 (49.9)				
Widowed	777 (36.9)				546 (33.6)				231 (48.0)				
Separated	52 (2.5)				43 (2.6)				9 (1.9)				
Education, n (%)													0.04
≤ Junior high school and others	977 (46.4)				732 (45.0)				245 (50.9)				
High school	933 (44.3)				733 (45.1)				200 (41.6)				
≥ Some college	198 (9.4)				162 (10.0)				36 (7.5)				
Current smoking, n (%)													0.002
No	2054 (97.4)				1595 (98.0)				459 (95.4)				
Yes	54 (2.6)				32 (2.0)				22 (4.6)				
Alcohol drinking, n (%)													<0.0001
No	1695 (80.4)				1278 (78.6)				417 (86.7)				
Yes	413 (19.6)				349 (21.5)				64 (13.3)				
Dietary supplement use, n (%)													0.0002
No	1475 (70.0)				1106 (68.0)				369 (76.7)				
Yes	633 (30.0)				521 (32.0)				112 (23.3)				
Physical activity, total metabolic equivalents-hours/d	38.1	34.3	-	43.2	39.6	35.9		44.1	32.9	30.9		36.4	<0.0001
History of chronic disease^d^, n (%)													<0.0001
No	1059 (50.2)				864 (53.1)				195 (40.5)				
Yes	1049 (49.8)				763 (46.9)				286 (59.5)				
Depression symptoms^e^, n (%)													<0.0001
No	1617 (76.7)				1363 (83.8)				254 (52.8)				
Yes	491 (23.3)				264 (16.2)				227 (47.2)				

*IQR* interquartile range

^a^Values are medians and interquartile ranges or numbers of subjects (%)

^b^Frailty score (0–5) was defined as the sum of poor physical function (two points), exhaustion (one point), low physical activity (one point), and unintentional weight loss (one point). A score of ≥3 was classified as indicating frailty

^c^Continuous values were compared by Mann–Whitney signed-rank test and proportions for categorical values were compared by the chi-square test

^d^History of chronic disease was defined as having any of the following self-reported disease: stroke, myocardial infarction, hypertension, diabetes, or chronic rheumatism

^e^Depression symptoms were defined as a Center for Epidemiologic Studies Depression score ≥16

**Table 2:** Energy and protein intakes and dietary TAC of 2108 old Japanese women categorized by no frailty and frailty^a^

	Total (*n =* 2108)				No frailty^b^ (*n =* 1627)				Frailty^b^ (*n =* 481)				*P* ^c^
	Median	IQR			Median	IQR			Median	IQR			
Energy, kcal/d	1693	1396	-	2012	1729	1425	-	2042	1589	1317	-	1905	<0.0001
Total protein, g/d	73.1	65.0	-	81.4	73.6	65.5	-	82.1	70.6	63.4	-	79.4	0.0001
Total protein, % energy	16.7	14.7	-	18.8	16.9	14.9	-	18.9	15.8	14.2	-	18.2	<0.0001
Total protein, g/kg BW/d	1.43	1.22	-	1.67	1.45	1.23	-	1.68	1.40	1.19	-	1.66	0.045
Animal protein, g/d	42.0	33.7	-	51.8	42.7	34.1	-	52.3	39.8	32.9	-	49.6	0.004
Animal protein, % energy	9.5	7.4	-	11.9	9.7	7.6	-	12.0	8.9	7.0	-	11.3	<0.0001
Plant protein, g/d	30.3	27.7	-	33.2	30.3	27.9	-	33.3	29.9	27.1	-	32.8	0.02
Plant protein, % energy	7.0	6.4	-	7.6	7.0	6.4	-	7.6	6.9	6.2	-	7.7	0.10
Dietary TAC, mmol TE/d	20.2	15.7	-	25.0	20.9	16.5	-	26.1	18.2	13.6	-	22.2	<0.0001
Dietary TAC, mmol TE/1000 kcal	11.7	9.0	-	15.1	12.0	9.4	-	15.4	10.7	7.8	-	13.9	<0.0001

*BW* Body wight, *IQR* interquartile range, *TAC* total antioxidant capacity, *TE* Trolox equivalent

^a^Values are medians and interquartile ranges

^b^Frailty score (0-5) was defined as the sum of poor physical function (two points), exhaustion (one point), low physical activity (one point), and unintentional weight loss (one point). A score of ≥3 was classified as indicating frailty

^c^Values were compared by Mann–Whitney signed-rank test and proportions for categorical values were compared by the chi-square test

Total protein intake was significantly inversely associated with frailty (*P* for trend = 0.001), and a similar association was observed in animal protein intake (*P* for trend = 0.04) (Table 3). These associations were maintained after further adjustment for dietary TAC (*P* for trend = 0.003 for total protein and 0.03 for animal protein). Meanwhile, no association was observed between the intake of plant protein and frailty (*P* for trend = 0.30). Although a weak inverse association was observed in the second tertile in the adjustment for animal protein, further adjustment of dietary TAC attenuated the association. Dietary TAC was also significantly inversely associated with frailty in multivariate adjusted model (*P* for trend < 0.0001). After further adjustment for intake of each protein, the association between dietary TAC and frailty were maintained (All *P* for trend < 0.0001). The associations between total protein and frailty in the adjustment for dietary TAC and between dietary TAC and frailty in the adjustment for total protein were examined by using one regression model. The multivariate adjusted ORs (95% CIs) in the third tertile compared by the first tertile were 0.66 (0.49, 0.87) for total protein and 0.52 (0.39, 0.71) for dietary TAC. The association of dietary TAC was higher than that of total protein.

**Table 3:** Multivariate adjusted odds ratios and 95% confidence intervals for frailty compared to no frailty by tertile of dietary total antioxidant capacity and protein among 2108 old Japanese women^a^

	T1 (Lowest) (*n =* 702)	T2 (Intermediate) (*n =* 703)	T3 (Highest) (*n =* 703)	*P* for trend
Total protein^b^, g/d	≤67.6	67.6–78.3	>78.3	
Frailty^c^, %	28.5	20.8	19.2	
Model 1^d^	1.00 (ref)	0.61 (0.46, 0.80)	0.64 (0.48, 0.84)	0.001
Model 1^e^ + dietary TAC^f^	1.00 (ref)	0.62 (0.46, 0.82)	0.66 (0.49, 0.87)	0.003
Animal protein^b^, g/d	≤36.9	36.9–48.4	>48.4	
Frailty^c^, %	25.9	22.3	20.2	
Model 1^e^	1.00 (ref)	0.78 (0.59, 1.03)	0.75 (0.57, 1.00)	0.04
Model 1^e^ + dietary TAC^f^	1.00 (ref)	0.76 (0.57, 1.01)	0.75 (0.56, 0.99)	0.04
Model 1^e^ + plant protein^f^	1.00 (ref)	0.75 (0.57, 1.00)	0.68 (0.51, 0.92)	0.01
Model 1^e^ + plant protein + dietary TAC^f^	1.00 (ref)	0.75 (0.56, 1.00)	0.71 (0.53, 0.96)	0.03
Plant protein^b^, g/d	≤28.6	28.6–32.0	>32.0	
Frailty^c^, %	25.6	21.3	21.5	
Model 1^e^	1.00 (ref)	0.78 (0.59, 1.03)	0.86 (0.65, 1.14)	0.30
Model 1^e^ + dietary TAC^f^	1.00 (ref)	0.82 (0.62, 1.09)	0.99 (0.74, 1.33)	0.91
Model 1^e^ + animal protein^f^	1.00 (ref)	0.72 (0.54, 0.97)	0.76 (0.57, 1.03)	0.08
Model 1^e^ + animal protein + dietary TAC^f^	1.00 (ref)	0.77 (0.58, 1.03)	0.89 (0.65, 1.21)	0.42
Dietary TAC^b^, mmol TE/d	≤17.3	17.3–23.1	>23.1	
Frailty^c^, %	30.6	23.6	14.2	
Model 1^e^	1.00 (ref)	0.80 (0.61, 1.04)	0.51 (0.38, 0.69)	<0.0001
Model 1^e^ + total protein^f^	1.00 (ref)	0.82 (0.63, 1.08)	0.52 (0.39, 0.71)	<0.0001
Model 1^e^ + animal protein^f^	1.00 (ref)	0.80 (0.61, 1.05)	0.51 (0.38, 0.68)	<0.0001
Model 1^e^ + plant protein^f^	1.00 (ref)	0.79 (0.60, 1.04)	0.51 (0.37, 0.69)	<0.0001

*CI* confidence interval, *OR* odds ratio, *ref* reference, *TAC* total antioxidant capacity, *TE* Trolox equivalent

^a^Values are ORs (95%CIs), unless otherwise indicated

^b^Protein intakes and dietary TAC were energy-adjusted according to the residual method

^c^Frailty score (0–5) was defined as the sum of poor physical function (two points), exhaustion (one point), low physical activity (one point), and unintentional weight loss (one point). A score ≥3 were classified as frailty

^e^Adjusted for age (y, continuous), body mass index (kg/m2, continuous), residential block (Hokkaido and Tohoku, Kanto, Hokuriku and Tokai, Kinki, Chugoku and Shikoku, or Kyushu), size of residential area (city with a population ≥1 million, city with a population <1 million, or town and village), living alone (yes or no), current smoking (yes or no), alcohol drinking (yes or no), dietary supplement use (yes or no), history of chronic disease (any of stroke, myocardial infarction, hypertension, diabetes, or chronic rheumatism; yes or no), depression symptoms (yes or no), and energy intake (kcal/d, tertiles)

^f^Further adjusted for total protein (g/d, tertiles), animal protein (g/d, tertiles), plant protein (g/d, tertiles), or dietary TAC (mmol/TE, tertiles)

The subjects were divided nine groups based on the combination of the tertile of total protein intake and the tertile of dietary TAC, and the risk of frailty was predicted in these nine groups (Table 4). The group composed of the highest tertile for both total protein intake and dietary TAC (P3A3) had a markedly low prevalence of frailty. The multivariate adjusted OR (95% CIs) for frailty in P3A3 was 0.27 (0.16, 0.44) (*P* = 0.0001) compared with the reference group of the lowest tertile for both total protein intake and dietary TAC (P1A1).

**Table 4:** Multivariate adjusted odds ratios and 95% confidence intervals for frailty compared to no frailty based on a combination of total protein and dietary total antioxidant capacity among 2108 old Japanese women^a^

	Total protein^b^, g/d			
	P1 (Lowest)	P2 (Intermediate)	P3 (Highest)	*P*_Protein_ for trend
	≤67.6	67.6–78.3	>78.3	
Dietary TAC^b^, mmol TE/d				
A1 (Lowest) ≤17.3	(*n =* 273)	(*n =* 229)	(*n =* 200)	
Frailty^c^, %	38.5	23.6	28.0	
Model 1^d^	1.00 (ref)	0.42 (0.27, 0.65)	0.67 (0.43, 1.05)	0.03
A2 (Intermediate) 17.3–23.1	(*n =* 209)	(*n =* 238)	(*n =* 256)	
Frailty^c^, %	27.3	24.4	19.9	
Model 1^d^	0.62 (0.39, 0.96)	0.55 (0.36, 0.86)	0.47 (0.29, 0.76)	0.22
A3 (Highest) >23.1	(*n =* 220)	(*n =* 236)	(*n =* 247)	
Frailty^c^, %	17.3	14.4	11.3	
Model 1^d^	0.47 (0.29, 0.76)	0.33 (0.20, 0.53)	0.27 (0.16, 0.44)	0.03
*P*_TAC_ for trend	0.001	0.56	0.002	

*CI* confidence interval, *OR* odds ratio, *ref* reference, *TAC* total antioxidant capacity, *TE* Trolox equivalent

^a^Values are ORs (95% CIs), unless otherwise indicated

^b^Protein intake and dietary TAC were energy-adjusted according to the residual method

^c^Frailty score (0–5) was defined as the sum of poor physical function (two points), exhaustion (one point), low physical activity (one point), and unintentional weight loss (one point). A score of ≥ 3 indicated frailty

^d^Adjusted for age (y, continuous), body mass index (kg/m^2^, continuous), residential block (Hokkaido and Tohoku, Kanto, Hokuriku and Tokai, Kinki, Chugoku and Shikoku, or Kyushu), size of residential area (city with a population ≥ 1 million, city with a population < 1 million, or town and village), living alone (yes or no), current smoking (yes or no), alcohol drinking (yes or no), dietary supplement use (yes or no), history of chronic disease (any of stroke, myocardial infarction, hypertension, diabetes, or chronic rheumatism; yes or no), depression symptoms (yes or no), and energy intake (kcal/d, continuous)

We also examined the association between FRAP, TEAC, or TRAP and frailty. Similar results to Tables 3 and 4 were confirmed (data not shown).

Dietary intake and dietary TAC were described among the subject of P1A1, P2A2, and P3A3, respectively (Table 5). For many food intakes, e.g., pulses, potatoes, fruits, vegetables, fish and shellfish, meats, eggs, and dairy products, positive associations were observed in the order of P1A1, P2A2, and P3A3. Meanwhile, the negative associations were obtained for rice, confectionaries, and soft drinks. The intakes of almost all the nutrients examined and dietary TAC were increasing according to increasing of protein intake and dietary TAC. Only the carbohydrate intake was inversely associated with the increasing of protein intake and dietary TAC among all nutrients.

**Table 5:** Comparison of dietary intakes and dietary total antioxidant capacity between the women of the lowest tertile (P1A1), the intermediate tertile (P2A2) and highest tertile (P3A3) for both protein intake and dietary total antioxidant capacity^a^

	P1A1 (*n =* 273)				P2A2 (*n =* 238)				P3A3 (*n =* 247)				*P* ^b^
	Median	IQR			Median	IQR			Median	IQR			
Energy intake, kcal/d	1709	1423	-	2077	1575	1297	-	1905	1700	1405	-	2032	0.004
Food intake^c^, g/d													
Rice	368.7	292.7	-	423.5	277.4	227.4	-	353.4	207.0	147.0	-	256.0	<0.0001
Noodles	43.3	26.3	-	70.9	48.1	32.8	-	67.0	43.9	23.3	-	75.5	0.25
Bread	27.0	15.0	-	54.1	27.3	12.4	-	58.7	27.3	12.8	-	56.5	0.64
Pulses	58.1	39.7	-	74.7	77.6	57.2	-	108.0	102.2	71.2	-	123.1	<0.0001
Potatoes	40.4	25.7	-	66.9	50.1	32.0	-	76.4	57.8	40.2	-	97.8	<0.0001
Confectioneries	65.0	45.8	-	95.7	56.8	39.4	-	79.6	43.1	23.5	-	62.8	<0.0001
Fruits	65.5	40.2	-	113.8	97.9	65.8	-	142.3	127.6	79.1	-	186.8	<0.0001
Total vegetables	227.1	172.7	-	288.8	289.8	239.4	-	360.2	404.7	297.0	-	511.0	<0.0001
Green tea	170.9	41.6	-	380.0	428.3	369.6	-	607.0	562.2	379.6	-	609.4	<0.0001
Black and oolong tea	9.1	−3.2	-	23.7	14.7	3.0	-	41.0	25.6	5.4	-	117.0	<0.0001
Coffee	21.3	1.9	-	117.6	59.5	14.0	-	148.5	157.0	105.0	-	365.7	<0.0001
Soft drinks	7.9	−2.2	-	21.6	7.6	−1.0	-	16.1	4.5	−4.6	-	15.7	0.03
Fish and shellfish	68.6	49.8	-	83.9	95.7	80.2	-	111.3	142.3	113.5	-	178.0	<0.0001
Meats	41.5	28.3	-	55.5	57.7	43.5	-	75.4	63.7	43.6	-	83.4	<0.0001
Eggs	32.1	21.7	-	48.2	37.1	26.3	-	54.6	45.8	29.6	-	65.7	<0.0001
Dairy products	103.4	37.5	-	153.0	128.9	69.7	-	173.5	155.8	91.2	-	199.0	<0.0001
Nutrient intake^c^													
Protein, g/d	61.6	56.8	-	64.8	73.1	70.1	-	75.5	86.8	82.1	-	93.4	<0.0001
Animal protein, g/d	31.6	26.2	-	36.1	42.0	38.9	-	45.4	56.4	50.8	-	61.9	<0.0001
Plant protein, g/d	29.6	27.7	-	31.6	30.3	28.0	-	33.3	31.1	28.5	-	33.9	<0.0001
Fat, g/d	44.2	38.9	-	49.9	50.5	45.4	-	55.5	55.0	48.6	-	61.1	<0.0001
Marine origin n-3 polyunsaturated fat^d^, g/d	0.80	0.60	-	1.02	1.13	0.92	-	1.37	1.63	1.31	-	2.09	<0.0001
Carbohydrate, g/d	267	254	-	280	243	230	-	254	219	204	-	234	<0.0001
Total dietary fiber, g/d	11.7	9.7	-	13.2	13.3	11.9	-	15.3	16.4	14.0	-	19.3	<0.0001
β-carotene, μg/d	2882	2100	-	3902	3861	2992	-	5242	5246	3948	-	7704	<0.0001
Vitamin D, μg/d	12.0	8.8	-	14.9	17.9	15.1	-	21.1	28.2	21.4	-	34.9	<0.0001
Vitamin C, mg/d	104	82	-	132	148	123	-	177	188	158	-	225	<0.0001
Sodium, mg/d	3872	3427	-	4278	4269	3900	-	4658	4981	4525	-	5465	<0.0001
Potassium, mg/d	2129	1871	-	2465	2754	2526	-	3051	3564	3194	-	4005	<0.0001
Calcium, mg/d	476	393	-	562	623	539	-	691	820	708	-	951	<0.0001
Magnesium, mg/d	219	198	-	236	266	253	-	292	342	312	-	378	<0.0001
Iron, mg/d	7.0	6.2	-	7.7	8.9	8.1	-	9.6	10.9	9.8	-	12.1	<0.0001
Dietary TAC^c^, mmol TE/d	13.0	10.0		15.3	20.1	18.9		21.7	27.5	24.9		31.8	<0.0001

*IQR* interquartile range, *P1A1* the lowest tertile for both total protein intake and dietary TAC group, *P2A2* the intermediate tertile for both total protein intake and dietary TAC group, *P3A3* the highest tertile for both total protein intake and dietary TAC group, *SD* standard deviation, *TAC* total antioxidant capacity, *TE* Trolox equivalent

^a^ Values are median and interquartile range

^b^ Values were compared by Kruskal-Wallis test

^c^ Energy-adjustment was performed according to the residual method

^d^ Sum of eicosapentaenoic acid, docosapentaenoic acid, and docosahexaenoic acid

All the results shown in Tables 3, 4 and 5 were obtained using dietary variables adjusted by the residual method. Similar results were observed for the density method (data not shown).

## Discussion

In the present study, a higher intake of total and animal protein and dietary TAC were independently associated with a lower prevalence of frailty among old Japanese women. Further, the prevalence of frailty was markedly low in the subjects who consumed a diet with both high total protein and high dietary TAC. Such individuals had a significantly greater intake of pulses, potatoes, fruits, vegetables, fish and shellfish, meats, eggs, and dairy products and a lower intake of rice, confectionaries, and soft drinks than did those with both low total protein intake and low dietary TAC. To our knowledge, this is the first study to investigate the association of protein intake and dietary TAC with frailty, not only independently but also cooperatively.

Japanese government recommends daily total protein intake for old generation aged ≥70 years of 0.85 g/kg BW [30]. However, the present study showed that total protein intake was 1.45 g/kg BW/d for non-frail group. Even in the frail group, the respective value was 1.40 g/kg BW/d. Previous review studies showed that some study described the daily protein intake of 0.8 g/kg BW/d is insufficient for the maintenance of muscle mass and proposed 1.0–1.5 g/kg BW/d among old population [10, 12]. Although we cannot adequately discuss the appropriate amount of protein intake in this study due to the limited validity of the BDHQ, the amount of protein required to maintain muscle mass for old population might be higher than the present recommendation in Japan.

Median (IQR) dietary TAC among our subjects was 20.2 (15.7–25.0) mmol TE/d. Our previous study showed that median (IQR) dietary TAC among young Japanese women estimated by the comprehensive diet history questionnaire, on which the BDHQ was based for development, was 16.8 (12.4–24.1) mmol TE/d [31]. Although these values could not be compared directly, dietary TAC among the present participants might be higher than that of young Japanese women in the previous study.

Although the essential biological mechanism that causes frailty has never been adequately explained, hypotheses have proposed that the loss of muscle mass may be one of the causes of frailty [9–12, 32] and that sufficient dietary protein intake was required to maintain muscle mass and function [10, 12]. The inverse association of dietary protein with frailty in previous studies [14–17] may be caused by preventing loss of muscle mass or improving the synthesis of muscle protein. Meanwhile, inflammation and oxidative stress, which also cause the reduction of muscle protein synthesis and promotion of muscle proteolysis, may play an important role in the development of frailty [11, 33, 34]. The inverse association between the intake of antioxidant nutrients and frailty in previous studies [14, 18] may be explained by the restriction of inflammation. Our results showed that both protein intake and dietary TAC were inversely associated with frailty. These associations were consistent in the previous studies [14–18], and observed independently they may suggest that dietary protein and antioxidant activity individually prevent frailty by maintaining muscle mass and function.

Plant protein was not associated with frailty in our present study, albeit the association was observed in our previous study [13]. Although these studies were conducted using the same dataset, the previous study used quintiles instead of tertiles to categorize dietary intake leading more extreme group. This different methodological approach may cause different result. Our additional investigation using bisection, quartile, and quintile showed that, in only the quintile, plant protein was associated with frailty (data not shown). These different results may indicate that the effect of plant protein on frailty is relatively weak. The weak inverse association between plant protein and frailty in the adjusted model using animal protein was attenuated after further adjustment of dietary TAC. Many food sources of plant protein, e.g. pulses and vegetables, contributed to dietary TAC in this population [13, 19], and the correlation between dietary TAC and plant protein (0.24) was higher than that between dietary TAC and total protein (0.07) or animal protein (-0.03) in the present study. The effect of plant protein on frailty observed in the previous study [13] may have been caused by the antioxidant nutrients included in plant foods rather than the protein. In fact, our additional analysis showed that the significant inverse association between plant protein and frailty using quintile was disappeared after further adjustment for dietary TAC (data not shown).

In our study, the prevalence of frailty in the group with P3A3 was lowest among the groups. This association was more marked than those of single high protein and dietary TAC values, indicating that a diet containing both high protein and high antioxidant nutrients has the potential to prevent frailty more effectively than does high protein or high antioxidants solely. Although almost all of the combinations of the tertiles of total protein and dietary TAC were showed lower ORs than P1A1, only P3A1 showed non-significant association. The reason was unclear. This result might imply that the inverse association between protein and frailty was relatively weak under the low intake level of antioxidants. The previous studies showed that Mediterranean [35–37] and prudent dietary patterns [38] were associated with a low prevalence of frailty. This association may be caused by an abundance of both protein and antioxidants derived from fruits, vegetables, whole cereals, and oily fish. Not only increasing the intake of protein or antioxidants individually, but also increasing both of them simultaneously may be effective for frailty prevention.

The present subjects in the P3A3 group had higher intake of pulses, potatoes, fruits, vegetables, fish and shellfish, meats, eggs, and dairy products and lower intakes of rice, confectionaries, and soft drinks than did those in the P1A1 group. The P3A3 subjects ate more of almost all the nutrients, except for carbohydrates, than did P1A1 subjects. Avoiding confectionaries or soft drinks and eating more fruits, vegetables, pulses, and fish and shellfish may be an effective dietary strategy for preventing frailty in the present population. Drinking green tea or coffee, which are the main contributors of dietary TAC in old Japanese women [19], instead of soft drinks, may be another way to prevent frailty. Appropriate food selections to increase the intake of protein and dietary TAC, based on food culture and dietary habits of target populations, may be important in frailty prevention.

The strength of our present study was our ability to examine the relation of protein intake and dietary TAC with frailty in a large number of old women using multicenter epidemiological data. The subjects lived over a wide geographical range of Japan and had various dietary and lifestyle habits. Additionally, the dietary questionnaire used has been validated [20, 21].

Several limitations of this study also warrant mention. First, dietary TAC was only moderately associated with plasma TAC measurements in previous studies [39, 40], and the method of evaluating the total antioxidant function *in vivo* is controversial [41]. However, several studies have demonstrated that the consumption of antioxidant-rich foods increased plasma TAC immediately after ingestion [42]. Furthermore, previous studies showed that dietary TAC was inversely associated with inflammatory molecules [43, 44]. Although the validity of dietary TAC estimated by the BDHQ has not been examined, dietary TAC estimated by a comprehensive diet history questionnaire, from which the BDHQ was developed, was also inversely associated with a serum inflammatory marker in our previous study [31]. These results may suggest that dietary TAC is a useful tool for assessing antioxidant intake and antioxidant activities *in vivo* [41, 45]. Second, we used the score of the physical functioning scale of the SF-36 as a surrogate for walking speed and grip strength. However, all the criteria we used to define frailty were very similar to those proposed by Woods et al. [5], who showed that the physical functioning scale dichotomized at the 25th percentile was strongly associated with poor walking speed and moderately associated with poor grip strength, and maintained that their definition predicted outcomes as well as did Fried’s definition [5]. These results may indicate the appropriateness of the criteria we used. Third, the BDHQ was a self-reported diet history questionnaire and are subject to both random and systematic measurement errors as all the other self-reported dietary assessment methods. To minimize the effect of misreporting, we excluded the subject reporting low or high energy intake and we used energy-adjusted values. Fourth, because reliable food composition table for dietary supplements could not be obtained in Japan, we could not consider the intake of dietary supplements in calculating nutrient intake and dietary TAC. However, we used the variable for dietary supplement use (yes or no) as confounders. Fifth, the present study was conducted under a cross-sectional design, which prevents the investigation of a causal effect of protein intake or dietary TAC on frailty. Therefore, we tried to minimize the effect of reverse causality by excluding subjects assumed to be under restricted protein intake (chronic kidney disease) or who had a disability (Parkinson’s disease or those who were unable to walk), and also by calculating ORs adjusted for the history of chronic disease. The proportion of the subjects with these diseases is assumed to be underestimated because of self-reported, which is a further limitation of this study. Meanwhile, we examined the food source of protein among the subjects categorized by no frailty and frailty. The contribution of fish was significantly lower for frailty (29%) than for no frailty (30%) and the contribution of animal food was significantly lower (57% vs 58%) and plant food was higher (44% vs 42%) for frailty than for no frailty. These differences were small and the contributions of meat, dairy products, and eggs were not significantly different between the groups. Frail participants might not avoid eating to meat and similar food source of protein was obtained between frail and non-frail group, may indicate that there might be no problem of reverse causality for the cause of masticatory problems. Sixth, almost all subjects in the present study were grandmothers of selected dietetic students, and not a random sample of old Japanese women. Not all Japanese adolescents enter college or university (enrollment ratio: 57%) [46], and the grandmothers of students who do so might accordingly have a relatively high social and economic status. Further, the nutrition interest of their grandchildren might influence their dietary habits. Thus, our results cannot be readily extrapolated to the general old Japanese population. Finally, although we attempted to adjust for a wide range of potential confounding variables, we were unable to rule out residual confoundings. Additionally, we should have excluded subjects with poor cognitive function because poor cognition is related to frailty [47] and might be associated with dietary TAC [48]. Since our self-reported questionnaires did not examine cognitive function, we could not exclude subjects with poor cognition. However, the study subjects answered the questionnaires themselves, which implies sufficient cognitive function to do so. Meanwhile, cognitive problems could also lead to unreliable answers to the questionnaires.

## Conclusions

We found that total protein intake and dietary TAC was independently inversely associated with frailty in old Japanese women. The diet with the combination of high total protein and high dietary TAC was markedly associated with a low prevalence of frailty. Eating fruits, vegetables, pulses, and fish and shellfish and drinking green tea and coffee, instead of confectionaries and soft drinks, may be an effective strategy for frailty prevention among the Japanese population. In other populations, other food combinations that allow for an increase of both protein and antioxidants in their diet can be selected based on the local food culture and dietary habits. Further studies are needed to develop effective dietary strategies for the intervention of frailty prevention.

## Abbreviations

BDHQ: Brief-type self-administered diet history questionnaire
BMI: Body mass index
BW: Body weight
CI: Confidence interval
FRAP: Ferric reducing ability of plasma
IQR: Interquartile range
MET: Metabolic equivalent
OR: Odds ratio
ORAC: Oxygen radical absorbance capacity
P1A1: The lowest tertile for both total protein intake and dietary total antioxidant capacity
P2A2: The intermediate tertile for both total protein intake and dietary total antioxidant capacity
P3A3: The highest tertile for both total protein intake and dietary total antioxidant capacity
SF-36: Short-form 36-item health survey
TAC: Total antioxidant capacity
TE: Trolox equivalent
TEAC: Trolox equivalent antioxidant capacity
TRAP: Total radical-trapping antioxidant parameter
